# Therapeutic Potential of Targeting the Ghrelin Pathway

**DOI:** 10.3390/ijms18040798

**Published:** 2017-04-11

**Authors:** Gustav Colldén, Matthias H. Tschöp, Timo D. Müller

**Affiliations:** 1 Institute for Diabetes and Obesity & Helmholtz Diabetes Center, Helmholtz Zentrum München German Research Center for Environmental Health (GmbH), 85764 Neuherberg, Germany; gustav.collden@helmholtz-muenchen.de (G.C.); matthias.tschoep@helmholtz-muenchen.de (M.H.T.); 2 Division of Metabolic Diseases, Department of Medicine, Technische Universität München, 80333 Munich, Germany; 3 Institute for Diabetes and Obesity (IDO), Business Campus Garching-Hochbrück, Parkring 13, 85748 Garching, Germany

**Keywords:** ghrelin, therapy, pathology, inflammation, anorexia

## Abstract

Ghrelin was discovered in 1999 as the endogenous ligand of the growth-hormone secretagogue receptor 1a (GHSR1a). Since then, ghrelin has been found to exert a plethora of physiological effects that go far beyond its initial characterization as a growth hormone (GH) secretagogue. Among the numerous well-established effects of ghrelin are the stimulation of appetite and lipid accumulation, the modulation of immunity and inflammation, the stimulation of gastric motility, the improvement of cardiac performance, the modulation of stress, anxiety, taste sensation and reward-seeking behavior, as well as the regulation of glucose metabolism and thermogenesis. Due to a variety of beneficial effects on systems’ metabolism, pharmacological targeting of the endogenous ghrelin system is widely considered a valuable approach to treat metabolic complications, such as chronic inflammation, gastroparesis or cancer-associated anorexia and cachexia. The aim of this review is to discuss and highlight the broad pharmacological potential of ghrelin pathway modulation for the treatment of anorexia, cachexia, sarcopenia, cardiopathy, neurodegenerative disorders, renal and pulmonary disease, gastrointestinal (GI) disorders, inflammatory disorders and metabolic syndrome.

## 1. Introduction

**Figure 1:**
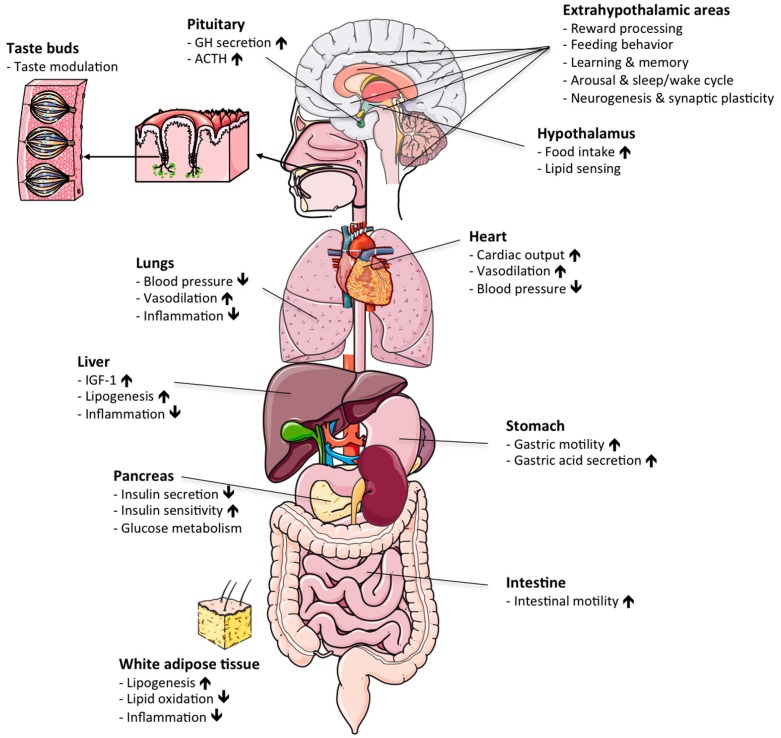
Physiological actions of ghrelin: schematic illustrating the various hormonal actions of ghrelin in different target organs. ACTH, adrenocorticotropic hormone. GH, growth hormone. IGF-1, insulin-like growth factor 1. Up/down arrows denote increase/decrease.

In the early 1980s, Cyril Bowers and Frank Momamy generated a group of opioid peptide derivatives that potently stimulated the release of growth hormone (GH) from the anterior pituitary [1,2]. Of appreciable note, these GH-releasing peptides affected GH release independently of the growth-hormone-releasing hormone (GHRH) pathway [3,4,5,6] and also activated neuronal populations in the hypothalamic arcuate nucleus [3]. Together, these data pointed to the presence of an as-yet unknown endocrine system with metabolic action not only on the pituitary, but also on the hypothalamus. In 1996, Roy Smith and Lex van der Ploeg identified the receptor for the GH-releasing peptides as a full-length seven-transmembrane G-protein-coupled receptor, which henceforth became known as the growth hormone secretagogue receptor 1a (GHSR1a) [7]. In 1999, Masayasu Kojima and Kenji Kangawa identified the gastrointestinal peptide hormone ghrelin as the endogenous ligand of GHSR1a [8]. Initially identified as an endogenous GH secretagogue, work by Matthias Tschöp and Mark Heiman soon later identified ghrelin as a neuropeptide with strong ability to enhance food intake and appetite via stimulation of CNS neurocircuitry [9]. While ghrelin remains as of today the only known peripheral gut hormone that is capable of stimulating food intake and body weight gain, a myriad of studies have subsequently revealed that ghrelin is a hormone with a vast spectrum of physiological actions that go far beyond its initial classification as a hunger hormone (Figure 1; for a review, see [10]). In this regard, beyond its ability to stimulate food intake via centrally-regulated signal mechanisms [11,12,13], ghrelin acts on the stomach to enhance gastric acid secretion and gastric motility [14], modulates taste sensation [15], stress and anxiety [16], sleep/wake cycle [17], glucose metabolism [18] and brown fat thermogenesis [19]. Due to these and numerous other beneficial effects on systems metabolism, pharmacological modulation of the endogenous ghrelin system is widely considered a promising approach to treat a variety of metabolic complications, most prominently gastroparesis and pathological underweight associated with anorexia nervosa or cancer cachexia. In this review, we will summarize the various biological actions of ghrelin, with a special focus on the druggability of the endogenous ghrelin system for the aforementioned diseases.

## 2. Molecular Structure and Synthesis of Ghrelin

**Figure 2:**
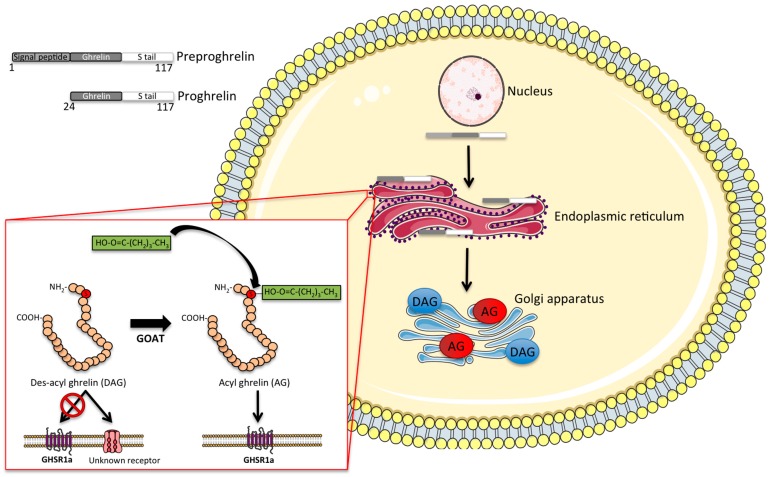
Intracellular processing of ghrelin: schematic illustrating the intracellular processing of ghrelin. The 117-aminoacid precursor peptide preproghrelin is cleaved to proghrelin, which is in turn cleaved by prohormone convertase 1/3 to the mature 28-amino acid ghrelin peptide. Prior to secretion, des-acyl ghrelin is acylated by the hormone ghrelin-*O*-acyltransferase (GOAT), which permits its binding to the growth-hormone secretagogue receptor 1a (GHSR1a). AG, acyl ghrelin. DAG, des-acyl ghrelin.

Ghrelin is a 28-amino acid peptide that is primarily produced and secreted by X/A-like cells in the oxyntic glands of the gastric mucosa, especially in the gastric fundus [8]. The *ghrelin* gene encodes the 117-amino acid precursor peptide preproghrelin, which is post-translationally cleaved to produce the mature 28-amino acid ghrelin peptide (Figure 2).

To activate its only known receptor, ghrelin requires attachment (acylation) of a fatty acid (preferably C8 or C10) to its serine 3 residue, a rare post-translational modification achieved by the enzyme ghrelin-o-acyltransferase (GOAT) [20,21]. GOAT is the only enzyme capable of acylating ghrelin in vivo, as indicated by the absence of acyl ghrelin in mice lacking GOAT [22]. The GOAT-mediated ghrelin acylation is necessary for ghrelin to bind and activate the GHSR [21]. However, although ghrelin acylation is required for most of the physiological effects of ghrelin, including the activation of orexigenic hypothalamic neurocircuits [23], some studies suggest a metabolic role also for des-acyl ghrelin. In detail, des-acyl ghrelin has been reported in some studies to affect feeding [24], adiposity [25] and glucose metabolism [26] by mechanisms independent of GHSR signaling. However, some studies report that the des-acyl form of ghrelin rather antagonizes the effect of acyl ghrelin on feeding [27,28]. In addition, des-acyl ghrelin has been shown to affect differentiation of C2C12 muscle cells [29] and further shows some cardioprotective effects on the heart [30], supposedly acting via an as-yet unidentified receptor [31]. In the general circulation, acyl ghrelin is rapidly degraded by various esterases, such as carboxylesterase and butyrylcholinesterase [32]. The half-life of acyl ghrelin accordingly varies between 10 and 30 min in rodents [32,33] and up to 4 h in humans [32].

The ghrelin receptor is primarily expressed in brain, but lower levels can also be found in certain peripheral organs, such as pancreas, spleen, kidney and adrenal gland [34,35]. In rodent brain, GHSR expression is greatest in the pituitary and in the orexigenic neuropeptide Y/agouti-related protein (NPY/AgRP) neurons of the arcuate nucleus (ARC), but is also highly expressed in regions associated with reward processing, such as the substantia nigra and the ventral tegmental area (VTA) [36].

Ligand-induced activation of GHSR1a leads to an increase in intracellular calcium levels by the activation of G-protein subtype G_aq/11_, which stimulates the production of inositol triphosphate (IP3), activation of protein kinase C (PKC) and phospholipase C (PLC) with a concomitant release of calcium from the ER [37]. In addition, GHSR activation entails activation of G_i/o_ signaling mechanisms [38,39] and induces the phosphorylation of ERK1/2, as well as induction of the PI-3 kinase (PI3K) and phosphorylation of Akt [40,41,42]. In the hypothalamic NPY/AgRP neurons, ghrelin increases levels of the intracellular energy sensor AMPK [43]. AMPK switches off ATP-consuming pathways and turns on ATP-generating pathways, such as glucose uptake and fatty acid oxidation. Downstream actions of AMPK include the disinhibition of CPT1, which transports fatty acids into mitochondria for oxidation. In the hypothalamus, ghrelin increases the concentration of fatty acids, the substrate for mitochondrial fatty acid oxidation and concurrently increases expression of CPT1 to facilitate fatty acid oxidation [44]. Along this line, ghrelin potently activates the mitochondrial uncoupling protein 2 (UCP2), which functions as a free radical scavenger and whose stimulation is crucial for ghrelin-induced stimulation of NPY neurons and mitochondrial proliferation [45]. Ghrelin activation of UCP2 may thus be part of an endocrine mechanism to buffer the ghrelin-activated cells from excess ROS production by the fatty acid oxidation. This provides a potential mechanism whereby NPY/AgRP neurons can maintain a high ghrelin-induced firing rate during low energy states, whereas the anorexigenic pro-opiomelanocortin (POMC) neurons with their lack of GHSR receptor-induced UCP2 activation cannot buffer against ROS, as well, and thus, cannot maintain neuronal firing during starvation [45]. In addition, ghrelin was reported to act via the GHSR to activate the deacetylase Sirt1, which acts through the tumor suppressor gene p53 to increase AMPK levels [46]; blockade of this pathway abolishes the central orexigenic effects of ghrelin [47,48]. Associated with these signaling cascades is upregulation of several transcription factors, including Bsx, forkhead box O1 (FoxO1) and cAMP response element binding protein (pCREB) [49], as well as the mammalian target of rapamycin (mTOR) pathway [50].

While ghrelin was identified as a potent stimulator of GH release from the anterior pituitary [8], it soon became apparent that ghrelin is far more than a GH secretagogue. Accordingly, since its discovery in 1999, a myriad of preclinical and clinical studies have shown that ghrelin exerts a surprising variety of metabolic actions in such distinct areas as the regulation of food intake and satiety [51], modulation of energy expenditure via regulation of brown fat thermogenesis [19], regulation of lipid storage and utility, which is notably independent of ghrelin’s orexigenic effects [9], and modulation of the stress axis [52], sleep/wake rhythm [17], GI motility [14] and glucose metabolism [18].

The most well-established effect of ghrelin is the stimulation of food intake via the activation of orexigenic NPY/AgRP neurons in the ARC [11,12,13]. Although ghrelin may act in other brain regions important for feeding behavior, such as the mesolimbic reward circuits [53] or the brainstem [54], ghrelin does not stimulate feeding in mice lacking NPY/AgRP neurons, demonstrating that this population is essential for ghrelin-induced feeding [55]. Consistent with a role for ghrelin in regulating feeding behavior, circulating levels of acyl ghrelin rise shortly prior to anticipated meal time and return to baseline levels within the first hour after a meal [56,57,58,59,60]. While both total ghrelin levels and acyl ghrelin levels have been reported to progressively increase during fasting in pigs [61] and rodents [62], acyl ghrelin levels seem to be rather suppressed by long-term fasting in humans [60]. Such discrepancies may be due to different measurement methods, as Gahete and colleagues observed a fasting-induced increase in acyl ghrelin in mice using a commercial ELISA kit [62], whereas Kirchner and colleagues did not observe fasting-induced increase in acyl ghrelin as measured with MALDI-TOF mass spectrometry [63]. Suppression of acyl ghrelin during fasting would however be consistent with the observation that acylation of ghrelin relies predominantly on dietary rather than endogenously-derived lipids [63]. Notably, ghrelin enhances adiposity independently of its orexigenic effects by increasing lipid storage and by decreasing lipid utilization [9,64].

## 3. Ghrelin and Inflammation

Apart from its well-established orexigenic and lipogenic effects, ghrelin possesses anti-inflammatory effects and ameliorates anti-oxidative stress under a variety of pathological conditions. This was first demonstrated in a rat model of surgically-induced septic shock, where concurrent ghrelin treatment was shown to reduce the mortality rate by nearly 50% [65,66]. Soon thereafter, both ghrelin and its receptor were shown to be expressed in human immune cells, and ghrelin acts on both immune cells and endothelial cells to block the expression of pro-inflammatory cytokines induced by inflammatory stimuli such as leptin, TNF-α and endotoxins [67,68]. Research since then has clearly established the anti-inflammatory actions of ghrelin and has shown its potential in the treatment of a multitude of inflammatory disorders. In a rat model of arthritis, daily treatment with the ghrelin analog GHRP-2 for eight days ameliorated arthritis scores and abolished the effects of arthritis on the hypothalamic-pituitary-adrenal (HPA) axis [69]. Ghrelin abolishes the inflammation, weight loss, diarrhea and abnormal colonic histology in mice and rats with trinitrobenzene-induced colitis [70,71]. Ghrelin similarly prevents inflammation, oxidative stress and organ damage associated with pancreaticobiliary inflammation [72] and several other inflammatory conditions, such as chronic airway respiratory infection [73], experimental autoimmune encephalomyelitis [74] and diabetic neuropathy [75]. Beneficial effects of ghrelin have also been demonstrated in several conditions of acute inflammation. Twice-daily ghrelin injections for one week were shown to ameliorate deleterious post-infarct inflammatory myocardial remodeling caused by coronary artery ligation in rats [76]. Furthermore, ghrelin completely reversed the histological and biochemical alterations caused by experimentally-induced subarachnoid-hemorrhage in rats, normalizing plasma cytokine levels and brain oxidative stress markers, which was associated with improved neurological scores 48 h after hemorrhage, [77]. Similar effects of ghrelin, including enhanced seven-day survival, were observed in rats with cerebral ischemia induced by middle cerebral artery occlusion [78]. Finally, both the acylated and desacylated forms of ghrelin have been shown to reverse the pro-inflammatory effect of chronic high-fat diet feeding [79,80,81,82].

Despite a plethora of animal studies supporting the therapeutic anti-inflammatory potential of ghrelin, only a few human trials have been conducted on the use of ghrelin in inflammatory disorders. Kodama and colleagues reported that three-week administration of ghrelin to patients with chronic respiratory infection reduced neutrophil density and air sputum inflammatory cytokine levels, as well as increased exercise tolerance [73]. In a randomized double-blind placebo-controlled trial, three weeks of ghrelin treatment in patients with COPD similarly improved respiratory symptoms and respiratory strength [83]. More recently, a randomized trial showed ghrelin treatment to reduce inflammation and pulmonary complications during the postoperative period following esophagectomy [84].

## 4. Ghrelin GI Motility

One of the earliest established functions of ghrelin is its effect on the GI tract. In particular, Masuda and colleagues showed as early as 2000 that ghrelin stimulates both gastric motility and gastric acid secretion in rats, effects that were notably abolished by bilateral vagotomy [14]. In addition, ghrelin stimulates fasting-like gastric motor activity, likely via a pathway involving the stimulation of NPY neurons, preganglionic dorsal vagal complex (DVC) neurons and vagal afferent neurons [85,86,87], but also via direct action on the GI tract through intrinsic cholinergic neurons in the small intestine [88]. The ability of ghrelin to stimulate gastric motility was later confirmed also in human subjects [89]. Induced gastrointestinal dysfunction is associated with impaired ghrelin signaling [90], and ghrelin levels are found to be reduced in multiple GI disorders, such as gastritis [91], *H. pylori* infection [92], dyspepsia and reflux disease [93,94]. Ghrelin has further been shown to improve gastric emptying in multiple rodent models of dyspepsia and gastroparesis [95,96,97,98,99], suggesting its therapeutic potential for the treatment of GI disorders. In humans, ghrelin treatment of patients with dyspepsia increased food intake and subjective appetite [100], while a randomized clinical trial in gastroparesis patients showed ghrelin to relieve gastroparesis symptoms, including nausea and vomiting [101]. Treatment with the ghrelin signaling potentiator rikkunshito was further shown to improve symptoms of dyspepsia, associated with an increase in plasma ghrelin levels [102]. Finally, relamorelin (also known as RM-131), a selective GHSR agonist [103], was shown to substantially improve symptoms of delayed gastric emptying in a randomized phase 1b trial of diabetic patients [104] and is currently in a clinical phase 2b trial for the treatment of diabetic gastroparesis [10,105].

## 5. Anorexia/Cachexia

Anorexia is defined as pathological underweight associated with the loss of the desire to eat. Anorexia can be associated with psychological disorders, such as anorexia nervosa, or with chronic organic diseases that cause a loss of appetite, such as chronic obstructive pulmonary disease (COPD), acquired immunodeficiency syndrome (AIDS), end-stage cancer or sepsis. In the most severe cases, anorexia co-exists with cachexia, which is defined as excessive skeletal muscle and adipose tissue wasting as a result of a chronic imbalance between anabolic and catabolic processes. Cachexia is typically characterized by an abnormal release of pro-inflammatory cytokines and activation of the sympathetic nervous system (SNS), both important factors contributing to the pathologically-negative energy balance that is typically observed in patients with cachexia. Due to its orexigenic and lipogenic action, ghrelin is commonly acknowledged to possess therapeutic potential for the treatment of anorexia and cachexia (for a review, see [106]).

There are several preclinical and clinical studies supporting a role for ghrelin in the treatment of pathological underweight and cachexia. In this regard, treatment with ghrelin or ghrelin analogs has been shown to increase food intake and body weight in several rodent models of anorexia/cachexia, even though partial resistance to the effects of ghrelin has sometimes been observed in rodent models of cachexia [107,108]. In rats, ghrelin treatment suppresses the cachectic weight loss induced by human melanoma cancer cell implantations [109]. Improved food intake and prevention of tissue wasting has further been observed upon ghrelin treatment in a mouse model of lung cancer-induced cachexia [110], as well as in a series of studies evaluating the role of ghrelin and its analogs in rodent tumor-bearing models of cachexia [108,111,112,113,114]. In line with these data, ghrelin stimulates feeding and lean body mass accrual in a rat model of chronic kidney disease-induced cachexia [115], attenuates cachexia in rats with heart failure [116], inhibits skeletal muscle breakdown after burn injury in rats [117] and inhibits angiotensin II-induced cachexia in mice [118].

In humans, a number of studies have demonstrated that ghrelin levels are elevated in patients suffering from anorexia nervosa [119,120,121,122,123] and cachexia [124,125] and that the ability of ghrelin to acutely stimulate subjective hunger is blunted in anorexia nervosa patients [126], which raised the possibility that ghrelin action is limited in these disorders. However, other studies demonstrated that long-term treatment with ghrelin can indeed stimulate hunger and feeding in patients with anorexia nervosa [127]. Other than the duration of treatment, the discrepant results may be explained by the mode of administration, which varied between the different studies from continuous ghrelin infusion [126] to twice daily 5-min i.v. infusion [127]. Most recently, it was shown that intranasal administration of the ghrelin receptor agonist GHRP-2 for one year was effective in improving appetite, body weight and glycemia in a severely emaciated anorexia nervosa patient [128]. There is by now also numerous human trials showing that ghrelin and ghrelin agonists can effectively improve anorexia and cachexia in cancer patients with few adverse effects [129,130,131]. Of special interest is the recently developed ghrelin receptor agonist anamorelin, which holds promise for the treatment of cancer anorexia-cachexia [132,133,134,135]. Similarly, several studies report that chronic ghrelin treatment improves appetite in patients suffering from renal failure [136,137].

## 6. Cardiovascular Disorders

Ghrelin and its receptor are both expressed in the rodent and human heart [138,139], and ghrelin is known to exert effects on the cardiovascular system in rodents and healthy human subjects [140,141]. Soon after its discovery, ghrelin was shown to improve cardiac function in a rat model of heart failure [116]. Ghrelin has also been shown to prevent cardiac hypertrophy and fibrosis after myocardial infarction [142] and protects against ischemia/reperfusion injury in the rat heart [143]. Consequently, ghrelin treatment has been found to reduce mortality in rodent models of cardiovascular disease [144]. Ghrelin may exert its protective effect on the heart through multiple pathways, including activating AMPK and autophagy [145,146], as well as modulating ER stress [147].

Although there is scarce data on the use of ghrelin for cardiovascular disorders in human subjects, it was shown that intravenous administration of ghrelin for three weeks reduces arterial blood pressure, improves left ventricular function, work capacity and peak oxygen consumption during exercise in patients with chronic heart failure [116,148], as well as reversing endothelial dysfunction in patients with metabolic syndrome [149], suggesting its therapeutic potential for human patients suffering from cardiovascular and circulatory disorders.

## 7. Sarcopenia

Sarcopenia refers to the degenerative loss of skeletal muscle mass, commonly associated with aging, but may also accompany cachexia more generally. Ghrelin levels are generally found to be lower in elderly compared to younger subjects [150,151,152,153], and elderly subjects with sarcopenia have lower ghrelin levels than those without sarcopenia [153].

In rodents, ghrelin has been found to prevent muscle-wasting associated with tumors and cisplatin treatment [154], and enhancing ghrelin signaling with the ghrelin-potentiators rikkunshito was found to inhibit age-related sarcopenia and to prolong survival in a rodent model of senescence [155]. Chronic central ghrelin treatment may also enhance bone mass in rodents [156].

In humans, a randomized double-blind placebo-controlled cross-over trial found that oral administration of the ghrelin mimetic ibutamoren (MK-677) for 12 months in healthy elderly subjects increased GH and IGF-1 levels to that of younger adults and prevented lean mass loss without severe side effects [157].

## 8. Renal Failure

In mice, ghrelin levels are sharply increased by removal of the kidneys, and numerous studies in humans have shown ghrelin levels to be elevated in end-stage renal disease and chronic kidney failure, which likely reflects an impaired renal clearance of ghrelin [158,159,160,161,162]. Nonetheless, treatment with ghrelin has been shown to improve renal function and attenuate renal fibrosis and inflammation in rodent models of kidney disease or injury [163,164,165]. To date, no human data on the use of ghrelin to treat kidney disease are available.

## 9. Neurodegenerative Disorders

Aside from its central effects on hunger and appetite, ghrelin signaling plays a role in numerous higher brain functions, such as arousal and sleep-wake cycle [17,166], as well enhancing memory, learning, synaptic plasticity [16,167,168,169] and neurogenesis [170]. Ghrelin may in addition have a neuroprotective function in both hypothalamic and cortical neurons by inhibiting apoptosis [171,172,173] and inflammation [174]. Since ghrelin levels generally decline with age and exert a positive effect on memory, learning and neuroplasticity, the role of ghrelin in etiology and possible treatment of neurodegenerative disorders has garnered some interest (for extensive reviews, see [175,176]).

### 9.1. Dementia and Alzheimer’s Disease

Although it was initially reported that plasma levels of ghrelin were not changed in patients with Alzheimer’s disease [177], Gahete and colleagues later reported that several components of the ghrelin system, including ghrelin, GOAT and GHSR, were reduced in the temporal gyrus in the brain of patients with Alzheimer’s disease [178]. Although there are no reports on genetic association between ghrelin gene polymorphisms and AD [179], it was later reported that ghrelin gene variants are associated with cognitive dysfunction [180]. However, the exact nature of the relationship between ghrelin and cognitive function in healthy subjects is still unclear, since plasma ghrelin levels have been reported to rather be inversely associated with cognitive function in elderly non-demented patients [181]. Additionally, a more recent study in healthy young subjects concluded that, in contrast to the animal data, acute ghrelin administration did not enhance any parameters of cognitive function, including memory, mental speed or attention [182].

More specifically pertaining to Alzheimer’s disease, ghrelin has been shown to enhance insulin sensitivity and reduce abnormal tau phosphorylation of hippocampal neurons [183], as well as modulate the production of inflammatory cytokines induced by β-amyloid fibrils in microglia [184]. Central intracerebroventricular (ICV) administration of ghrelin ameliorated the cognitive dysfunction and neurodegeneration induced by intrahippocampal injections of amyloid-β oligomers in mice and rats, with associated improvements in cerebral glucose metabolism [185,186]. Oral administration of the ghrelin agonist LY444711 similarly improved cognition and reduced cerebral inflammation and beta-amyloid levels in the APP-SwDI genetic mouse model of AD (APP-Swedish K760N/M671L, Dutch E693Q and Iowa D694N mouse) [187,188]. Finally, peripheral administration of ghrelin rescued the impaired neurogenesis occurring in the 5XFAD (five familial AD mutations) genetic mouse model of AD [189]. However, in contrast to the abundant rodent studies, there are to date no trials on the effects of ghrelin in human Alzheimer’s or dementia patients.

### 9.2. Parkinson’s Disease

Interest in the relation between ghrelin and Parkinson’s began with the observation that ghrelin could protect striatal dopaminergic neurons, which express the GHSR, from degeneration caused by the neurotoxin 1-methyl-4-phenyl-1,2,3,6-tetrahydropyridine (MPTP) [190], possibly by preventing the activation of microglia [191]. Andrews and colleagues elaborated these results by showing that knocking out the ghrelin receptor in striatal neurons aggravated the neurotoxic effects of MPTP treatment and that the neuroprotective effects further involve enhanced mitochondrial biogenesis and UCP2-mediated reduction of reactive oxygen species [192]. The ability of ghrelin to protect against mitochondrial dysfunction and degeneration of dopaminergic neurons was demonstrated also in cells [146]. Most recently, ghrelin was shown to be essential to the neuroprotective effects of caloric restriction in the MPTP model of PD [193], an effect that requires the acylated (active) form of ghrelin [193]. PD often presents with gastrointestinal symptoms, such as delayed gastric emptying [194]. Ghrelin was shown to effectively treat symptoms of delayed gastric emptying in a rat model of PD utilizing the neurotoxin 6-hydroxydopamine [195]. In humans, it was shown that PD patients had an impaired postprandial ghrelin response [196], but no trials on the therapeutic potential of ghrelin for neurological or gastrointestinal aspects of PD have been performed to date.

### 9.3. Multiple Sclerosis

Multiple sclerosis (MS) is a disease characterized by progressive demyelination of neuronal axons. Neuroinflammation is central to the pathophysiology of MS [197], and given the anti-inflammatory and neuroprotective actions of ghrelin, which include protection against demyelination and axonal loss [198], it may have therapeutic potential for MS. Patients with MS have been found to have higher levels of ghrelin in the cerebrospinal fluid [199] and sometimes, but not always, in plasma [200,201], which may be interpreted as a protective response to the presence of neuroinflammation. MS may also be associated with genetic variation in the gene encoding the ghrelin receptor [202].

In several rodent models of experimental autoimmune encephalomyelitis (EAE), which is often used as a model for human MS, ghrelin has been shown to reduce the disease severity and associated neuroinflammation [74,203]. However, no clinical data with ghrelin in MS patients are currently available.

### 9.4. Amyotrophic Lateral Sclerosis

Amyotrophic lateral sclerosis (ALS) is a progressive neurodegenerative disease affecting mainly motor neurons in the brain, brainstem and spinal cord. The pathophysiology of ALS includes several metabolic alterations, which may interact with ghrelin signaling, such as loss of appetite [204], as well as neuroinflammation and reactive astrogliosis [205]. Despite ALS patients generally exhibiting lower BMI than healthy controls, several investigators have reported substantially lower ghrelin levels in ALS patients [206,207], suggesting that impaired ghrelin signaling may contribute to the disease progression. In line with this notion, ghrelin has shown neuroprotective effects on spinal cord motor neurons against glutamate-induced cell death in vitro, likely acting at least in part via the ERK/PI3K/GSK pathway, and inhibition of microglia [208,209]. Finally there is some preliminary evidence suggesting that ghrelin can slow down the disease progression of ALS in a mouse model [210], highlighting the need for further investigation of this relation in subsequent clinical trials.

## 10. Pulmonary Disease

Studies on the ghrelin system in pulmonary disease started with observations that ghrelin levels were increased in cachexic lung cancer patients, as well as underweight patients with COPD. These elevations were however likely unrelated to the lung pathology and rather secondary to the low BMI in these patients, since normal-weight subjects with either lung cancer or COPD had normal ghrelin levels [125,211,212,213,214]. Several studies even found decreased ghrelin levels despite underweight in COPD patients [215,216], although one recent study found that ghrelin levels were increased in patients with severe vs. moderate COPD, despite similar BMI [217]. A similar paradoxical phenomena with decreased ghrelin levels despite underweight was shown in cystic fibrosis patients [218], as well as tuberculosis patients [219,220], although other studies show normal or elevated ghrelin levels in cystic fibrosis, depending on disease severity [221].

A therapeutic potential of ghrelin for the treatment of lung cancer was suggested by the observation that GHSR expression is elevated in cancerous vs. normal lung tissue [222,223] and that ghrelin exerts anti-proliferative and pro-apoptotic effects on these cells in vitro [222], although a later study in a mouse lung cancer model was unable to show that ghrelin receptor agonism reduced the tumor growth progression [113].

In pulmonary hypertension, ghrelin levels have been found to be inversely associated with pulmonary arterial pressure [224]. In rodents, ghrelin has been shown to ameliorate the progression of pulmonary hypertension induced by monocrotaline or hypoxia and improve blood circulation [225,226,227,228], at least in part by preventing endothelial cell damage and maintaining NO release [229]. Ghrelin may also protect against hypoxia-induced lung injury by preventing hypoxia-induced increases in angiogenesis and HIF1-alpha and VEGF expression [230].

In rodents, ghrelin and ghrelin agonists have been shown to exert therapeutic effects, mainly by anti-inflammatory and anti-oxidative actions, and improve survival in several different models of lung injury. In three rat studies on sepsis-induced lung injury, sepsis induced by cecal ligation and puncture was shown to decrease ghrelin levels, and administration of ghrelin to restore plasma levels reduced the lung injury and inflammation, increased pulmonary blood flow and improved survival [231,232,233]. Similar effects were observed in rat models of lipopolysaccharide (LPS)-induced lung injury [234,235,236], pancreatitis-induced lung injury [237], paraquat-induced injury [238], bleomycin-induced injury [239] and lung contusion [240]. Ghrelin was also found to ameliorate the catabolic conditions and respiratory dysfunction induced by chronic cigarette smoking in rats [241], likely in part by inhibiting the pro-inflammatory effects of cigarette smoking in bronchial epithelial cells [242].

In humans, therapeutic effects of ghrelin and ghrelin agonists have been demonstrated for COPD. Kodama and colleagues showed ghrelin to suppress airway inflammation and increase functional exertion capacity in patients with chronic respiratory failure [73]. Miki and colleagues later showed that ghrelin enhanced exercise capacity, expiratory pressure and improved respiratory symptoms in COPD patients [83,243] and showed benefits on exertional dyspnea [244], These effects may be dose-dependent [245,246]. For lung cancer, treatment with the potent ghrelin analog anamorelin has shown benefits on appetite, lean mass retention, performance and quality of life [133,134,135].

## 11. Metabolic Disease

### 11.1. Obesity

Possibly the greatest interest in the therapeutic potential of ghrelin concerns its use for the treatment of obesity and metabolic syndrome, given its well-established role in stimulating appetite and inducing adiposity [9,247]. Conversely, it was established early on that antagonism of ghrelin signaling with the ghrelin antagonist d-Lys-3-GHRP-6 could reduce food intake and promote short-term weight loss or prevent weight gain in obese mice [248]. Results using knock-out models have however been inconsistent. Most studies indicate that deletion of ghrelin has no effect on growth, feeding behavior or susceptibility to diet-induced obesity, but at most might influence food preference [249,250,251]. However, one study shows that ghrelin-deficient mice may be protected from diet-induced obesity if HFD-feeding is initiated before adulthood [252]. In another study, mice lacking the ghrelin-acylating enzyme GOAT showed no phenotypic difference from wildtype mice when fed a standard high-fat diet, but did exhibit reduced fat gain and glucose intolerance when fed a high-fat/high-sugar diet [253]. Deletion of the GHSR seems to have a more convincing effect of lowering body weight and susceptibility to obesity [254,255,256], although some investigators found that GHSR deletion had no effect on these parameters [251,257].

In 2006, Zorilla and colleagues reported that vaccination of rats to produce anti-ghrelin antibodies results in decreased food efficiency and fat gain [258]. Thus, antagonism of ghrelin signaling was regarded as one of the most promising potential treatments for obesity. The first results along this line were published by Shearman and colleagues, using an RNA Spiegelmer to block ghrelin signaling in diet-induced obese mice for seven days, with which they achieved a two-gram weight loss in five days. Although ghrelin blockade transiently suppressed feeding, food intake rebounded to baseline within three days of treatment, and weight rebound was observed starting at four days of treatment, suggesting that ghrelin blockade induced a counter-regulatory response to prevent weight loss [259].

In 2007, the results of several different quinazolinone derivative-based GHSR antagonists were reported, which induced 10–15% weight loss in diet-induced obese (DIO) mice [260,261]. It was later reported that GHSR antagonism may also retard the development of ovariectomy-induced obesity in female mice [262]. More modest results have been demonstrated with GHSR inverse agonists [84] and GHSR fusion proteins [263]. Pharmacological inhibition of the GOAT enzyme was also shown to reduce food intake and weight gain in mice on a medium chain triglyceride (MCT)-rich high-fat diet [264]. However, in some studies, antagonism of ghrelin signaling did not affect feeding or long-term body weight [265,266], suggesting that there are compensatory mechanisms acting to restore homeostasis. Several such counter-regulatory responses to ghrelin antagonism that may act to promote a positive energy balance have been observed. For instance, Shearman and colleagues observed that inactivating plasma ghrelin with their ghrelin-binding RNA Spiegelmer caused a 15-fold increase in circulating levels of acyl ghrelin [259]. Gagnon and colleagues further observed that the prevention of HFD-induced weight gain obtained with a GHSR fusion protein reduced leptin gene transcription in visceral adipose tissue by as much as 90% [263]. An additional phenomena that may limit the potency of ghrelin signaling blockade is the ghrelin resistance that DIO mice are known to exhibit at both the peripheral and central level [267,268,269]. The limited efficacy of targeting ghrelin signaling in obesity has also been confirmed in humans: a randomized clinical trial with an anti-ghrelin vaccine demonstrated no additional weight loss compared to placebo, despite a clear induction of anti-ghrelin antibodies [270].

### 11.2. Prader Willi Syndrome

Prader Willi syndrome (PWS) is a genetic disorder characterized by numerous behavioral, neurological and physiological symptoms, chief among which are infertility, severe obesity and hyperphagia. Unlike subjects with simple and hypothalamic obesity [271], subjects with PWS also exhibit greatly elevated circulating ghrelin levels [272,273], which due to the hyperphagic pro-obesity effects of ghrelin was logically proposed as a potential cause of the hyperphagia and obesity observed in PWS. Current data do however not clearly support a causal relationship between ghrelin and obesity in PWS. A recent study reported that total ghrelin levels are substantially elevated in children with PWS long before any hyperphagia is evident, suggesting that elevated ghrelin levels may not be a major driving factor of obesity in PWS [274]. Consistent with this notion, acute treatment with the somatostatin analog octreotide was shown to greatly reduce circulating ghrelin levels in PWS subjects, but had no effects on body weight, body composition or resting energy expenditure [275]. A later randomized cross-over trial with octreotide for 16 weeks was shown to similarly reduce circulating ghrelin levels without affecting appetite or body mass [276]. However, since octreotide affects numerous metabolic hormones aside from ghrelin, trials with more selective ghrelin signaling antagonists in PWS are warranted. In addition, several studies have reported that abnormal feeding regulation and weight gain in PWS is more related to changes in the ratio of des-acyl ghrelin to acyl ghrelin than total levels per se [277,278], suggesting that GOAT inhibitors may be of particular interest for the treatment of PWS.

### 11.3. Diabetes and Hyperglycemia

Aside from hyperphagia and obesity, ghrelin is also extensively involved in the regulation of glucose metabolism [279]. Acyl ghrelin chiefly has hyperglycemic effects [280] and promotes insulin resistance [281,282], acting on both pancreatic islets to suppress insulin secretion [283] and on hepatocytes to stimulate glucose output [284], whereas des-acyl ghrelin may counteract the hyperglycemic effects of acyl ghrelin and enhance insulin sensitivity [282,284,285,286]. Consequently, there has been some interest in the potential of ghrelin antagonism to ameliorate diabetes and hyperglycemia. Several studies have shown that GHSR antagonists improve glucose tolerance associated with obesity by enhancing insulin secretion [261,287], and it was more recently demonstrated that ghrelin inhibition with the GHSR antagonist GHRP-6 could reverse diabetic symptoms also in a genetic mouse model of maturity-onset diabetes of the young, type 3 (MODY3) caused by mutations of the HNF1-alpha homeobox gene [288]. In humans, no clinical trials on the use of ghrelin signaling antagonists to treat hyperglycemia or diabetes have been performed to date.

### 11.4. Novel Approaches to Target the Ghrelin System in Metabolic Disease

A different approach to attempt to modulate the ghrelin system for therapeutic effect is to target either the cells producing ghrelin or the cells on which ghrelin acts. Using transgenic mice expressing the receptor for diphtheria toxin in ghrelin-secreting cells, it was shown that targeted ablation of ghrelin-secreting cells in adult mice with diphtheria toxin injections had no effect, not even transiently, on food intake, body weight or susceptibility to diet-induced obesity, despite reducing circulating ghrelin levels by 85–90% [289]. Altogether, to date, the evidence that blockade or antagonism of ghrelin signaling at either the peptide or receptor level could be an effective treatment against obesity must be considered weak, with effect sizes that even in the best cases are modest compared to treatments that target other gut hormones [290,291,292].

One intriguing alternative, to date untested, is to interfere with the overall activity of cells in the CNS that respond to ghrelin. In the CNS, the by far highest concentration of ghrelin receptors is found on the orexigenic NPY/AgRP neurons of the arcuate nucleus [293], which powerfully drive feeding when stimulated. These neurons are essential for normal feeding behavior, as evidenced by the rapid arrest of feeding and dramatic weight loss seen when these neurons are ablated in adult mice [294]. In obese leptin-deficient mice, AgRP neuron ablation was similarly observed to cause dramatic and sustained weight loss, along with normalization of glucose tolerance and fertility, demonstrating the potential of interference with this neuronal population to powerfully influence metabolic homeostasis [295]. Aside from the NPY/AgRP neurons, ghrelin is also known to act to a lesser extent in several other brain regions to influence feeding and hedonic behavior. Ghrelin can stimulate feeding, food reward and dopamine release in several regions associated with reward processing, such as the VTA and nucleus accumbens [53,167,296,297,298], as well as the amygdala [299], hippocampus [300] and dorsal vagal complex of the brainstem [54]. Drugs that target the activity of ghrelin receptor-expressing neurons might thus be expected to have a powerful and concerted effect on both homeostatic and hedonic feeding. Since recent studies have demonstrated the possibility to specifically target cells responding to a particular hormone by chemically hybridizing drugs to various gut hormones [292,301], a similar potential avenue for anti-obesity drugs might be to hybridize ghrelin to cytotoxic or neurotoxic drugs aiming to either permanently or reversibly block neuronal activity of ghrelin-targeted cells.

## 12. Concluding Remarks

**Figure 3:**
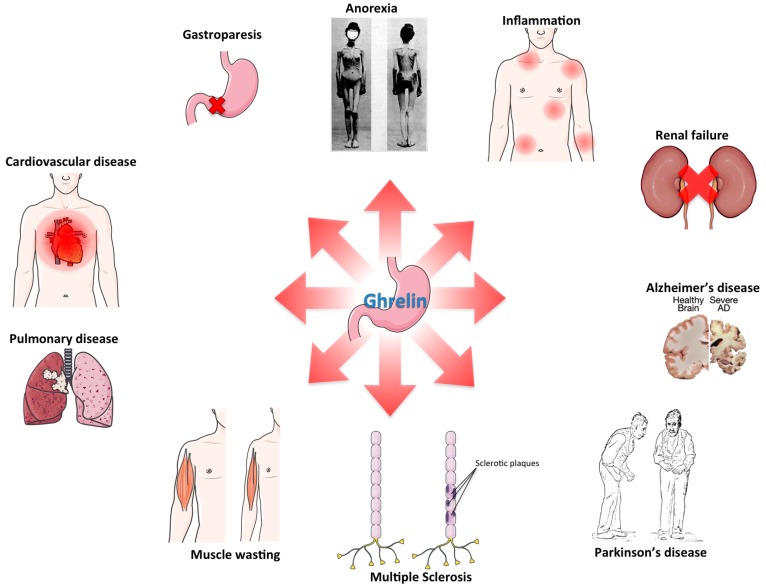
Avenues for ghrelin therapy: schematic of the various pathological conditions for which there is at least preclinical evidence suggesting potential therapeutic benefit of ghrelin treatment.

Although ghrelin was initially recognized for its role in feeding and metabolism, and consequently most expectations were pinned on its potential to treat obesity, the evidence to date, from rodent models of obesity, as well as human trials, suggests that antagonism of ghrelin signaling has only modest anti-obesity effects. Ghrelin is however an extremely pleiotropic hormone and may have therapeutic uses for a wide range of other diseases (Figure 3). In rodents, there is extensive evidence for the efficacy of ghrelin in a variety of inflammatory disorders, GI disorders, cardiovascular, renal and pulmonary disorders, as well as neurodegenerative diseases and wasting diseases. In humans, however, only a few of these potential treatments have so far been tested, leaving still wide room for investigations in the efficacy of ghrelin for the treatment of human disease.
